# A Simple Dual-Track HPLC-UV Methodology for Monitoring Primary Antiarrhythmic Drugs and Their Active Metabolites in Serum

**DOI:** 10.3390/ph19030406

**Published:** 2026-03-01

**Authors:** Paweł K. Kunicki, Wioleta Drózd, Jakub Meszka

**Affiliations:** Department of Drug Chemistry, Pharmaceutical and Biomedical Analysis, Medical University of Warsaw, Banacha 1, 02-097 Warsaw, Poland

**Keywords:** amiodarone, desethylamiodarone, propafenone, 5-hydroxypropafenone, N-depropylpropafenone, mexiletine, HPLC-UV, bioanalysis

## Abstract

**Objectives:** The aim of the work was to present a method for routine determination of antiarrhythmic drugs, propafenone (PPF), its two metabolites, 5-hydroxypropafenone (5OHPPF) and N-depropylpropafenone (NDPPF), mexiletine (MEX), amiodarone (AD) and desethylamiodarone (DEAD) in serum. **Methods:** A simple isocratic HPLC-UV system with a manual injector was applied. The separations were performed at ambient temperature on Supelcosil LC-CN column (150 × 4.6 mm, 5 μm). Two analytical procedures (A and B) were used: (A) for AD and (B) for PPF/MEX. The mobile phase for (A) was a mixture of: CH_3_OH:CH_3_CN:H_2_O:0.5M KH_2_PO_4_ (200:100:194:6 *v/v* + 0.1 mL 85% H_3_PO_4_ per 500 mL). The slightly acidified serum sample was extracted with hexane and the analytes were detected at 240 nm. The mobile phase for (B) was a mixture of: CH_3_CN:H_2_O:0.5M KH_2_PO_4_ (185:310:5 *v/v* + 0.1 mL 85% H_3_PO_4_ per 500 mL). The alkalized serum sample was extracted with diisopropyl ether, then back extracted into 0.01M HCl and finally the analytes were detected at 210 nm. **Results:** The method was calibrated with adequate selectivity and specificity in the range of 20–4000 ng/mL for AD, DEAD and MEX, 10–4000 ng/mL for PPF and 10–500 ng/mL for 5OHPPF and NDPPF. For all analytes, precision and accuracy fulfilled EMA requirements, i.e., ≤15% (≤20% for LLOQ), ensuring the reliability of the measurements. **Conclusions:** The method can be suitable for laboratories equipped with basic HPLC apparatus as an economical alternative to the LC-MS/MS technique.

## 1. Introduction

Drugs used to treat cardiac arrhythmias (antiarrhythmic drugs, AADs) carry a significant risk of side effects, and their pharmacokinetics are often complicated, making optimal dosing difficult. To improve this situation, attempts have been made for years to use therapeutic drug monitoring (TDM) for this purpose. However, the role of TDM for AADs is not as clear as, for example, for immunosuppressive drugs, aminoglycoside antibiotics or first-generation antiepileptic drugs. After initial hopes, reality verified the use of TDM in this group of drugs, which was influenced by both the decreasing frequency of their use resulting from the intensive development and implementation of non-pharmacological methods of treating cardiac arrhythmias, as well as the lack of clearly defined therapeutic ranges [1,2,3,4]. Despite these reservations and objections, TDM can be a useful tool, especially in therapy with digoxin and group I and III drugs (according to the Vaughan Williams classification) [3,4,5,6,7]. As new AADs appeared on the market, analytical methods enabling their monitoring in patients’ plasma/serum were published at the same time. Often, the chromatographic method also allowed for the determination of the main metabolite(s). These methods were intended for pharmacokinetic and TDM studies. An example is propafenone (PPF), a group Ic AAD, the use of which is complicated by polymorphic metabolism catalyzed by CYP2D6 [8], as well as amiodarone (AD) with the active metabolite DEAD associated with adverse effects such as pulmonary and tissue toxicity [5,6]. For AD, TDM is considered useful, although a clearly defined therapeutic range and supporting documentation from large clinical trials are lacking [2,4,7]. In addition to the numerous analytical methods for individual AADs and their metabolites, procedures for simultaneously measuring multiple AADs for TDM purposes have long been published [9,10,11,12,13,14,15]. More recent publications [11,13,14,15] use the mass spectrometry (MS), which is considered optimal, but not readily available everywhere (due to its cost). This is particularly true for low- and middle-income countries (LMICs). To support TDM and pharmacokinetic studies in these countries, there is a practical need for an economical and reliable analytical method enabling concentration monitoring of the primary AADs and their metabolites in patients’ blood. Most AADs (except digoxin) are present in patients at concentrations that allow the use of less specific than MS, but widely available UV detection coupled to relatively inexpensive HPLC equipment.

The aim of our work was to propose a useful procedure for routine determination of PPF, its two active metabolites, 5OHPPF and NDPPF, MEX, AD and DEAD in serum using the HPLC-UV technique, intended for laboratories where the unavailability of LC-MS/MS equipment may create a barrier limiting the analyses.

## 2. Results

### 2.1. Method Development

#### 2.1.1. Chromatographic Separation

A standard HPLC column with cyanopropyl packing (LC-CN) was selected for chromatographic separation, which has proven successful in many years of AAD bioanalysis [10,16]. For this type of stationary phase, a mobile phase based on a CH_3_CN-H_2_O mixture with adjusted ionic strength and moderate acidification is appropriate. Since AD and DEAD differ significantly in polarity from MEX and PPF (and PPF metabolites), gradient elution is indicated for separation. However, since AD and PPF/MEX are rarely administered simultaneously in clinical practice, it is more rational to perform two different separations using isocratic elution on the same column.

##### AD Mobile Phase

**Figure 1:**
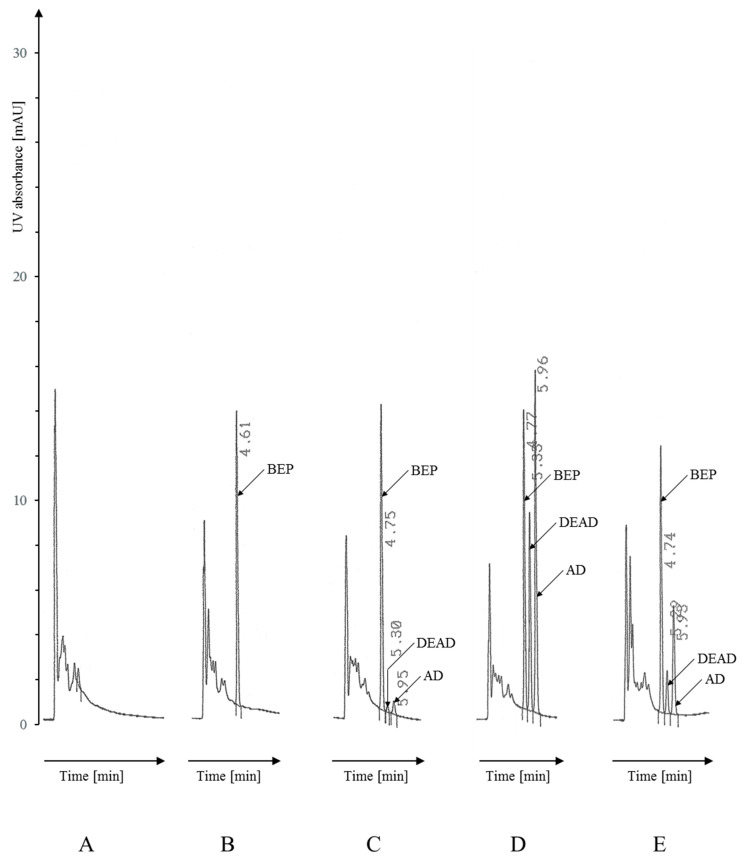
Chromatograms of extracted serum samples analyzed as described in Materials and Methods, Section 4.6.1. AD procedure (signal attenuation 32): (**A**) Drug-free serum analyzed without IS; (**B**) drug-free serum analyzed with IS; (**C**) drug-free serum spiked with AD/DEAD to obtain the concentration of 60 ng/mL (QC-L); (**D**) drug-free serum spiked with AD/DEAD to obtain the concentration of 1600 ng/mL (QC-M); (**E**) serum sample taken from the patient treated with AD containing 583 ng/mL of AD and 337 ng/mL of DEAD. Peaks: BEP (IS): 4.61–4.77 min, DEAD: 5.29–5.33 min, AD: 5.93–5.96 min.

Due to the significant difference in the solubility of AD and DEAD between CH_3_CN and CH_3_OH (in favor of CH_3_OH), for these analytes it was necessary to introduce CH_3_OH into the mobile phase and replace CH_3_CN in a 2:1 ratio. The mobile phase was experimentally selected as a mixture of: CH_3_OH:CH_3_CN:H_2_O:0.5M KH_2_PO_4_ (200:100:194:6 *v/v* + 0.1 mL 85% H_3_PO_4_ per 500 mL) pumped at a flow rate of 1.5 mL/min. The mobile phase composition ensured separation of AD, DEAD and the finally selected internal standard (IS); the decision on the choice of IS for AD/DEAD analysis is presented in Section AD Determination. Under the described conditions, satisfactory separation of analytes eluted at retention times (RT)—4.9 min (IS—bepridil (BEP)), 5.4 min (DEAD) and 6.1 min (AD)—was achieved, as well as no interference from the biological background after extraction from a serum sample (Figure 1). It should be emphasized that other substances considered/used as IS also eluted in satisfactory separation at RTs of 4.6 min, 5.0 min and 7.1 min for tamoxifen (TAM), dronedarone (DRO) and the AD analog L8040 (original IS), respectively.

##### PPF/MEX Mobile Phase

**Figure 2:**
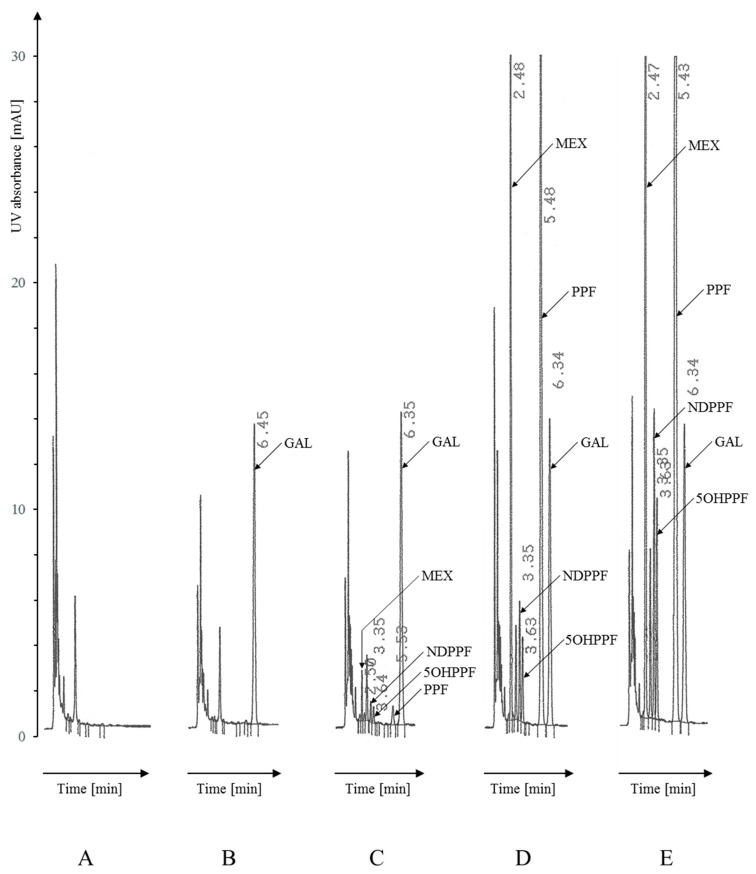
Chromatograms of extracted serum samples analyzed as described in Materials and Methods, Section 4.6.2. PPF/MEX procedure (signal attenuation 32): (**A**) Drug-free serum analyzed without IS; (**B**) drug-free serum analyzed with IS; (**C**) drug-free serum spiked with PPF/5OHPPF/NDPPF/MEX to obtain the concentration of 30/30/30/60 ng/mL (QC-L); (**D**) drug-free serum spiked with PPF/5OHPPF/NDPPF/MEX to obtain the concentration of 1600/160/160/1600 ng/mL (QC-M); (**E**) drug-free serum spiked with PPF/5OHPPF/NDPPF/MEX to obtain the concentration of 3200/400/400/3200 ng/mL (QC-H). Peaks: MEX: 2.47–2.50 min, NDPPF: 3.35 min, 5OHPPF: 3.63–3.64 min, PPF: 5.43–5.53 min, GAL (IS): 6.34–6.45 min.

For chromatographic separation for PPF/MEX analysis, a mobile phase with a simple composition was used, i.e., acetonitrile–water–0.5M KH_2_PO_4_. One of the goals of the presented method was to determine both active metabolites of PPF, i.e., 5OHPPF, regulated by CYP2D6, and the second, less pharmacologically active metabolite, NDPPF. The most complete separation of both PPF metabolites was achieved for the mobile phase: CH_3_CN:H_2_O:0.5M KH_2_PO_4_ (185:310:5 *v/v* + 0.1 mL 85% H_3_PO_4_ per 500 mL) resulting in a 0.32 min RT difference at a flow rate of 1.8 mL/min. For this purpose, both the percentage of acetonitrile and the ionic strength (obtained by the amount of added KH_2_PO_4_) were optimized. Reducing the percentage of acetonitrile in the mobile phase prolonged the RTs without significantly affecting the chromatographic separation. Reducing the phosphate content also prolonged the RTs and simultaneously improved the separation of NDPPF from 5OHPPF. Only at high concentrations, i.e., 500 ng/mL serum, unusual for a patient sample, is the separation incomplete, although sufficient for quantitative analysis. The selection of IS for PPF, 5OHPPF, NDPPF and MEX analysis is presented in Section PPF/MEX Determination. Under the presented conditions, satisfactory separation of analytes was obtained at RT of 2.5 min (MEX), 3.4 min (NDPPF), 3.7 min (5OHPPF), 5.6 min (PPF) and 6.5 min (IS—gallopamil (GAL)). No significant interference with biological background components was observed after serum sample extraction (Figure 2).

#### 2.1.2. Extraction

The procedure for isolating analytes from serum was a simple, standard liquid–liquid extraction. Automatic extraction was carried out using a Reax 2 rotary mixer, after confirming the appropriate process efficiency compared to manual extraction and after experimentally determining the optimal extraction time.

##### AD Procedure

For the isolation of AD/DEAD/BEP(IS) from the serum sample, hexane and a slightly acidic pH were selected, ensuring high yields for AD and BEP and sufficient yields for DEAD. Using a rotary mixer, the yields obtained were 92.0%, 56.4%, and 93.6% for AD, DEAD, and BEP, respectively, which was comparable to the recoveries for 4 min of manual extraction: 93.7%, 58.3%, and 92.4% for AD, DEAD, and BEP, respectively. For each analyte, the extraction efficiency was experimentally determined to remain stable for mixing times of 4–15 min, with the greatest variability occurring for the metabolite, likely due to its lower absolute recovery. Finally, it was decided that automatic extraction would be carried out for 6 min.

##### PPF/MEX Procedure

Suitable conditions for the extraction of PPF, its two metabolites, MEX and GAL (IS), are achieved by alkalizing the serum sample and using diisopropyl ether as the extractant. Diisopropyl ether is an efficient but not very selective reagent, often resulting in high background and numerous interferences in extraction from biological matrices. Therefore, to obtain a sufficiently pure sample, it is worth using a simple re-extraction step into a weak acid; this step does not significantly reduce the recovery of extracted analytes.

With this approach, the yields obtained for individual compounds after automated extraction compared to those obtained after 4 min of manual extraction were: for PPF, 72.1% vs. 74.76%, for 5OHPPF, 68.5% vs. 69.7%, for NDPPF, 68.2% vs. 69.7%, for MEX, 83.6% vs. 81.9%, and for GAL, 76.1% vs. 77.4%. Starting from a mixing time of 3 min, the analyte extraction efficiency was observed at a relatively constant level, comparable to the manual extraction efficiency. Therefore, 4 min was chosen as the optimal time to ensure satisfactory performance.

#### 2.1.3. Internal Standard Selection

Both for the determination of AD and for the PPF procedure, the following ISs were used years ago: L8040 and LU41616, respectively. These are analogs with similar physicochemical properties to the drugs being determined, which is a great advantage from the analytical point of view if they are available. As both of these compounds are no longer available (apart from the troublesome synthesis), it has become necessary to find alternative substances instead.

##### AD Determination

The problem of alternative IS for AD determination has been known for a long time and was thoroughly presented by Pollak, who after selecting candidate compounds proposed two—BEP and tamoxifen (TAM)—and finally choosing TAM [17]. In our studies, in addition to BEP and TAM, we also considered the antiarrhythmic drug dronedarone (DRO), which is structurally similar to AD. The obtained RTs and extraction yields (4 min manual extraction) are presented in Table 1. Each of these considered compounds can be accepted when analyzing the separation; signals from the biological matrix appeared at the latest for RT = 3.5 min, which determined the lack of interference. Efficiency was highest for BEP and the unavailable L8040 (89.9% and 88.7%, respectively) and slightly lower for TAM (83.9%). The extraction efficiency of 8.2% excluded DRO as a potential IS for the described method. Our results are similar to those of Pollak [17], but we prefer BEP as an IS because it is a drug currently not approved for treatment in the EU or the US, meaning that this substance (unlike TAM) will not be found in biological material collected from patients [17,18]. The above determined the final choice of BEP.

##### PPF/MEX Determination

The following substances were selected that could potentially be used as IS in the determination of PPF: diltiazem (DIL), flecainide (FLE), verapamil (VER), and GAL. The retention times of FLE (5.48 min) and PPF (5.80 min) were too close, resulting in incomplete separation. This problem was not observed for DIL, VER, and GAL. Both DIL and VER are frequently used in pharmacotherapy, which, in accordance with the requirements for compounds proposed as IS, significantly limits their potential application [18]. This does not apply to GAL, which is currently not registered as a drug. Further studies were conducted to confirm the GAL extraction efficiency using the analytical procedure for PPF. In a series of five samples extracted manually for 4 min followed by re-extraction into 0.01 M HCl, a satisfactory GAL extraction efficiency of 76.6% (72.8–83.2%) was obtained. It should be added that the analytical parameters of MEX make it possible to use this compound also as an IS if the method users wish to determine only PPF and its metabolites.

### 2.2. Method Validation

#### 2.2.1. Selectivity and Specificity

As mentioned earlier, the developed conditions allowed for the separation and elution of analytes in both the AD procedure and the PPF/MEX procedure. Sharp and symmetrical peaks were obtained at the following retention times: 4.9 min (BEP), 5.4 min (DEAD), and 6.1 min (AD) in the former procedure (Figure 1), and RTs of 2.5 min (MEX), 3.4 min (NDPPF), 3.7 min (5OHPPF), 5.6 min (PPF), and 6.5 min (GAL) in the latter (Figure 2). In accordance with EMA guidelines on validation, the study was conducted by analyzing 12 different serum samples not containing the tested substances, including one hemolyzed and one highly lipemic sample, prepared according to a given procedure [19,20]. For both separations, no significant interferences with serum-derived components were found; however, in the AD procedure, late elution of analytes is decisive, while in the PPF/MEX procedure, additional sample purification by re-extraction is crucial. Example chromatograms are shown in Figure 1 and Figure 2.

#### 2.2.2. Linearity, Calibration and Range

First, for each analyte, the linearity of the detection system was tested.

The detector response was easily described by linear equations:

AD: y = 270.4 x − 1303.2, r^2^ = 0.9999, (1)

DEAD: y = 283.7 x − 2049.5, r^2^ = 0.9999, and: (2)

PPF: y = 610.9 x − 556.3, r^2^ = 0.9998, (3)

5OHPPF: y = 473.5 x − 36.2, r^2^ = 0.9999, (4)

NDPPF: y = 661.6 x − 1435.7, r^2^ = 0.9997, (5)

MEX: y = 397.1 x − 1304.3, r^2^ = 0.9998, (6)

**Figure 3:**
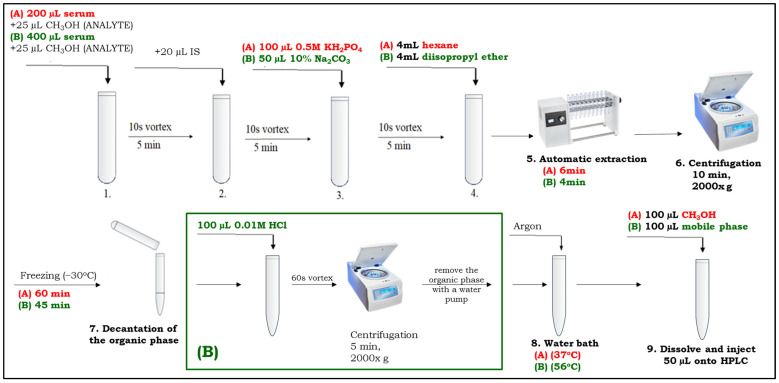
Sample preparation procedure as described in Section 4.6: (A) AD determination; (B) PPF/MEX determination.

The method was then calibrated in the ranges of 20–4000 ng/mL for both AD and DEAD, 10–4000 ng/mL for PPF, 10–500 ng/mL for both 5OHPPF and NDPPF, and 20–4000 ng/mL for MEX and found to be linear over the tested concentration range. Calibration curves (triplicates) were obtained by analyzing serum samples spiked with the analyte at each of the six tested concentration levels in duplicate. The determinations were performed at the concentrations presented for each analyte in Section 4.4 according to the two sample preparation procedures detailed in Section 4.6 and graphically presented in Figure 3. The curves were calculated by a weighted linear regression analysis with w = 1/x implemented for improving adjustment at low concentrations. The calibration curves after combining the measurements were described by the following equations:

F_AD_ = 0.000943 AD − 0.0004, r^2^ = 0.9993, (7)

F_DEAD_ = 0.000554 DEAD − 0.0022, r^2^ = 0.9836, (8)

and

F_PPF_ = 0.001470 PPF + 0.0016, r^2^ = 0.9991, (9)

F_5OHPPF_ = 0.001042 5OHPPF − 0.0020, r^2^ = 0.9974, (10)

F_NDPPF_ = 0.001265 NDPPF − 0.0016, r^2^ = 0.9953, (11)

F_MEX_ = 0.001009 MEX + 0.0036, r^2^ = 0.9982, (12)

where AD, DEAD, PPF, 5OHPPF, NDPPF and MEX stand for respective concentration in ng/mL, and F_AD(DEAD_,_PPF_,_5OHPPF_,_NDPPF_,_MEX)_ is a factor (ratio) obtained from peak areas: AD (DEAD, PPF, 5OHPPF, NDPPF, MEX)/IS.

The coefficient of determination obtained from the same samples showed a significant difference between the drug AD and its metabolite DEAD (r^2^ = 0.9993 and r^2^ = 0.9836, respectively). Analysis based on back-calculated concentrations showed that the AD calibration has a satisfactory precision (<10%) and low bias (<1.5%, and only 5% at LLOQ) whereas the DEAD calibration had a precision of 11–16% and a varying bias of up to 15% at five levels and 24% at LLOQ. This indicates DEAD extraction variability. The reliability of the calibration relationship for DEAD was later confirmed with accuracy and precision results meeting the established acceptance criteria.

#### 2.2.3. Precision and Accuracy

To evaluate the precision and accuracy of the method, the concentration of each analyte was measured at five levels, i.e., Lower Limit of Quantification (LLOQ), quality controls (QC): low (QC-L), medium (QC-M) and high (QC-H), Upper Limit of Quantification (ULOQ), as described in Section 4.4. Within-run and between-run evaluation was performed in five replicates.

Intra-assay precision, expressed as RSD, was <6.0%, <10.9%, <10.0%, <8.7%, <8.7%, and <8.2% for AD, DEAD, PPF, 5OHPPF, NDPPF, and MEX, respectively. Inter-assay precision, expressed by RSD, was <6.7%, <13.1%, <10.6%, <6.9%, <9.2%, and <7.3% for AD, DEAD, PPF, 5OHPPF, NDPPF, and MEX, respectively.

The accuracy for AD ranged from 95.3% to 103.9% for intra-run and from 96.9% to 103.8% for inter-run, for its metabolite DEAD from 91.4% to 105.5% intra-run and from 102.8% to 118.0% (at LLOQ) inter-run. The accuracy for PPF was from 97.9% to 101.4% for intra-run and from 99.0% to 117.9% (at LLOQ) for inter-run; for 5OHPPF from 93.7% to 104.8% intra-run and from 92.8% to 102.6% inter-run and for NDPPF from 93.9% to 112.7% intra-run and from 85.4% to 108.1% inter-run. The accuracy for MEX ranged from 89.8% to 102.8% for intra-run and from 96.5% to 101.9% for inter-run.

The detailed results are shown in Table 2. It can be noticed that DEAD differs in accuracy and precision from the other analytes, which is probably the result of less repeatable extraction; however, it should be emphasized that for all analytes, the results fulfilled EMA requirements regarding method precision and accuracy [19,20].

The analyte extraction yields obtained during precision and accuracy tests are presented in Table 3. The results confirmed good repeatability and recovery values similar to those obtained during method development. The extraction yields for the internal standards during the accuracy and precision tests were stable yielding 89.6 ± 2.2%, RSD = 2.48% and 80.3 ± 3.8%, and RSD = 4.73% for BEP and GAL, respectively.

#### 2.2.4. Other Validation Parameters

The LLOQ was assumed (according to EMA) to be the lowest calibration standard with acceptable accuracy and precision and was set at 10 ng/mL for PPF, 5OHPPF and NDPPF and at 20 ng/mL for AD, DEAD and MEX (Table 2). No carry-over was observed. Dilution integrity testing was intentionally omitted. During TDM, serum concentrations exceeding the ULOQ of 4000 ng/mL (AD, DEAD, PPF, MEX) and 500 ng/mL (PPF metabolites) are almost never observed in patients. Extremely rarely, this can occur immediately after intravenous administration of the drug.

## 3. Discussion

Many early HPLC methods for the determination of AD/DEAD were based on the use of the L8040 analog as IS; similarly, early methods for the determination of PPF and its metabolites used the LU41616 analog as IS. Thanks to the accumulated stock, these methods could be successfully used for some time after the possibility of obtaining these compounds had ceased. Later, the method had to be modified. Our method was successful in finding suitable ISs for both AD and PPF/MEX. We observed stability of IS working solutions for at least 6 weeks of use/storage. Both substances, BEP and GAL, are not currently used drugs, which makes them perfect ISs [18].

The presented methodology includes those AADs for which TDM can bring measurable therapeutic benefits; the method also includes their antiarrhythmically active metabolites (this is not the case with MEX). The literature contains information on therapeutic ranges discussed for AADs [2,7,15]. In relation to AD, despite criticism, monitoring the concentration of AD and DEAD allows for the reduction in underdosing and relatively dangerous overdosing, especially at high DEAD concentrations [3,4,6,7]. For PPF, monitoring serum concentrations of 5OHPPF and NDPPF alongside the parent drug allows for determination of the patient’s metabolic phenotype and dosing optimization correlated with CYP2D6 function [8]. For MEX, TDM may also be helpful in pharmacotherapy. Another AAD monitored in various cardiology centers is FLE; in our practice, given its infrequent use, there was no real demand for its measurement. However, it should be emphasized that with minor modifications to the mobile phase composition, the separation of FLE from PPF can be effectively improved, and since FLE is readily extractable in the PPF/MEX procedure, the FLE assay can be calibrated with minimal effort. Moreover, a simple methodology for monitoring a relatively new AAD, namely DRO and its active metabolite debutyldronedarone (DBD), using an identical LC-CN column was recently presented by our team [16].

The method presented here is characterized by validation parameters meeting the criteria for accuracy and precision included in the EMA guidelines [19,20]. The chromatographic properties of AD and DEAD determine that, under the developed separation conditions, there is practically no interference with matrix components or concomitant medications, all of which elute at RTs shorter than the analytes and IS. Such a favorable situation does not occur in PPF/MEX analysis. In this case, a simple re-extraction step into a weak acid is very helpful, resulting in a chromatographically pure sample with minimal risk of interference. Despite the nonspecific UV detection, the obtained precision and accuracy values within the calibrated concentration ranges demonstrate the reliability of the measurements. It should be emphasized that the calibrated ranges for individual analytes are fully adequate to the concentrations occurring during AD, PPF and MEX pharmacotherapy [2,7,15]. For AD and DEAD, an LLOQ of 20 ng/mL allows not only for the reliable determination of patient serum concentrations during therapy, but also for monitoring in the weeks to months following drug discontinuation. This LLOQ value is therefore more appropriate compared to those proposed in relatively recent publications, i.e., Juenke et al. (300 ng/mL) or Rodrigues et al. (100 ng/mL) [21,22]. The LLOQ value for PPF and its metabolites = 10 ng/mL also allows monitoring of each compound in patients with both extensive and poor CYP2D6 phenotype. The high ULOQ values of 4000 ng/mL for AD, DEAD, MEX, PPF and 500 ng/mL for 5OHPPF and NDPPF guarantee the measurement virtually in every patient sample collected. Exceptions would be cases of intentional intoxication or sampling shortly after intravenous administration. We can therefore conclude that the measurement ranges have been properly fitted to clinical practice.

Study limitations. The main limitation of the methodology presented in the manuscript is the lack of conventional stability studies required by the EMA validation guidelines [19,20]. This conscious decision stemmed from our focus on presenting the developed AAD separation and extraction conditions and the method’s capabilities to potential users. In our case, TDM conditions did not require repeated thawing of the sample (freeze–thaw test) or interruption of analyses (short-term stability), hence stability tests were not part of this particular study. The stability of AADs during storage is confirmed by results from various researchers, e.g., Slawson et al. recommended stability >4 weeks when frozen (MEX, AD, PPF); Rodrigues et al.: >30 days when frozen at −20 °C or −80 °C (AD, DEAD); Braakhuis et al.: >3 days at room temp., >7 days at 2–8 °C, also freeze–thaw (AD, DEAD, MEX); Li et al.: >30 days when frozen at −20 °C as well as freeze–thaw (AD, MEX) [11,13,15,22]. It is obvious that for the application of the literature method, in addition to confirming the accuracy and precision, it is necessary to check the stability in the local conditions of analysis and sample storage in your laboratory. Second, the method’s ranges were optimized for concentrations expected during routine TDM. Due to high ULOQ values, we waived the dilution integrity test, but consequently, the method is currently not recommended for use in clinical toxicology without supplementation of this test. Third, due to sporadic interference observed in the PPF/MEX analysis, interference studies with other co-administered drugs were not performed. Also, incurred sample reanalysis was not done. Fourth, the DEAD determination, despite meeting acceptance criteria, is characterized by lower precision and accuracy. We suggest that method reproducers take special care when calibrating this analyte.

The presented methodology is simple and economical. In addition to not employing MS/MS detection, the HPLC-UV instrumentation was simplified to a minimum. The pump is isocratic; it makes no sense to use gradient elution and analyze all compounds in a single run, as we wouldn’t expect to find several AADs simultaneously in a patient sample. Different chromatographic conditions for AD and PPF/MEX allow for the optimization of these separations and the selection of the optimal wavelength for UV detection. Using the same column and mobile phases very similar in ionic strength and pH means that the time required for chromatographic stabilization after mobile phase and flow change is only 15 min. The above also applies to our separately published method for determination of DRO and DBD [16]. We used a manual injector, which is sufficient for a series of several to a dozen or so samples. Clearly, using an autosampler instead will reduce laboratory workload and increase the attractiveness of the method. Our analyses were developed for ambient temperature; we did not use column thermostat (oven) which can improve separation if necessary. A stable temperature is essential for the repeatability of analyses and should be ensured, for example, by means of air conditioning. We use argon to evaporate the sample, but nitrogen will also perform the same role. The sample preparation shown graphically in Figure 3 is actually quite simple and does not take much time. It allows you to group samples into series, e.g., of eight or 16, and importantly, it uses only commonly available laboratory equipment, such as a vortex mixer, rotary mixer, laboratory centrifuge, freezer, water bath, etc. Our method has been successfully applied to the analysis of samples from patients with cardiac arrhythmias as part of routine AADs monitoring.

## 4. Materials and Methods

### 4.1. Chemicals

Chromatographic standards: AD hydrochloride (≥98%), DEAD hydrochloride (≥98%), BEP hydrochloride (≥98%), TAM (≥99%), PPF hydrochloride (≥98%), MEX hydrochloride (≥98%), and FLE acetate (≥98%) were obtained from Sigma-Aldrich (Steinheim, Germany); 5OHPPF hydrochloride (99.6%) and NDPPF hydrochloride (99.9%) were obtained from TLC PharmaChem (Newmarket, ON, Canada); DRO hydrochloride (99.99%) was from MedChemExpress, (Monmouth Junction, NJ, USA); GAL hydrochloride (≥98%) and VER hydrochloride (≥98%) were from Knoll (Ludwigshafen, Germany); DIL hydrochloride (≥98%) and L8040 hydrochloride (≥98%) were manufactured by Sanofi (Gentilly, France). Solvents and chromatographic reagents: HPLC-grade methanol, n-hexane, and 85% H_3_PO_4_ were obtained from J.T. Baker (Deventer, The Netherlands); HPLC-grade acetonitrile, diisopropyl ether, KH_2_PO_4_ (pro analysi), and Na_2_CO_3_ (pro analysi) were purchased from Merck (Darmstadt, Germany); HCl was from POCH S.A. (Gliwice, Poland). HPLC-grade water was obtained from water purification system (Millipore, Molsheim, France).

### 4.2. Instrumentation

A simple isocratic HPLC system (Spectra-Physics, San Jose, CA, USA) consisting of a P100 pump, an injector with 50 µL loop (model 7125i, Rheodyne, Cotati, CA, USA), a Spectra 100 UV detector, and a Chrom Jet 4400 integrator was used. Sample preparation and laboratory activities were performed using universal laboratory centrifuges: MPW 375 and MPW-260R (MPW, Warsaw, Poland), Reax 2 rotary mixer and Reax Top vortex mixer (Heidolph Instruments, Schwabach, Germany), water bath (LW 502, AJL Electronic, Krakow, Poland), PS-10A ultrasonic cleaner (CNC-Ultrasonic, Jedlnia Letnisko, Poland) and KL 1-02 universal laboratory pump (AGA LABOR, Warsaw, Poland). A Millipore Simplicity 185 Water Purification System was used to obtain HPLC grade water.

### 4.3. Chromatographic Conditions

A Supelcosil LC-CN column (150 × 4.6 mm, 5 µm) protected by a Supelguard LC-CN pre-column (20 × 4.6 mm, 5 µm) from Supelco (Bellefonte, PA, USA) was used for analyte separation. All separations were performed at ambient temperature, which was stabilized at 22 °C using air conditioning.

The mobile phase for AD determination: CH_3_OH:CH_3_CN:H_2_O:0.5M KH_2_PO_4_ (200:100:194:6 *v/v* + 0.1 mL 85% H_3_PO_4_ per 500 mL) was pumped at a flow rate of 1.5 mL/min. The applied conditions ensure the stability of elution of analytes in a series of tested samples (*n* = 25) with RSD: 0.70% (BEP), 0.81% (DEAD), 1.02% (AD). The analytical wavelength was set at 240 nm.

The mobile phase for PPF/MEX determination was a mixture of: CH_3_CN:H_2_O:0.5M KH_2_PO_4_ (185:310:5 *v/v* + 0.1 mL 85% H_3_PO_4_ per 500 mL). The flow rate was 1.8 mL/min. The elution stability of the analytes was confirmed in a series of tested samples (*n* = 25) with RSD: 0.71% (MEX), 0.36% (NDPPF), 0.40% (5OHPPF), 0.70% (PPF), 0.39% (GAL). UV detection was performed at a wavelength of 210 nm.

### 4.4. Stock and Working Solutions, Calibration Standards and Quality Controls

Stock solutions of AD, DEAD, BEP, DRO, L8040, PPF, MEX, 5OHPPF, NDPPF, GAL, VER, and DIL (1 mg/mL) were prepared by weighting and dissolving appropriate amounts of chemically pure hydrochlorides (acetate for FLE) of the substances (chemically pure substance for TAM) in methanol and then stored at 4 °C protected from light. Working solutions for the conducted tests and then for calibration standards (CAL) and control samples (QC) were prepared from stock solutions by appropriate dilution in methanol; once prepared, they were stored at 4 °C. Working solutions were later added to the drug-free serum to obtain the desired analyte content. Concentration levels for AD and DEAD were prepared as follows: 20, 80, 250, 800, 2000, 4000 ng/mL (calibration) and 60, 1600, 3200 ng/mL (quality controls: QC-L, QC-M, and QC-H, respectively). Concentration levels for PPF/5OHPPF/NDPPF/MEX were prepared as follows: 10/10/10/20, 50/25/25/100, 200/50/50/400, 800/100/100/1000, 2000/200/200/2000, 4000/500/500/4000 ng/mL (calibration) and of 30/30/30/60, 1600/160/160/1600, 3200/400/400/3200 ng/mL (quality controls: QC-L, QC-M and QC-H, respectively). BEP (IS for AD) working solution (20 µg/mL) and GAL (IS for PPF/MEX) working solution (10 µg/mL) were also prepared from their stock solutions by adequate dilution in methanol, then stored refrigerated at 4 °C and stable for a period of at least 6 weeks. The working solutions of both ISs ensured high concentration stability expressed by RSD = 1.44% (range: −1.85%, +3.14%; *n* = 25) and RSD = 0.68% (range: −1.03%, +1.27%; *n* = 20) for BEP and GAL, respectively.

### 4.5. Biological Matrix

Drug-free serum required for method development and validation was obtained from healthy donors (volunteers). The collected blood was centrifuged, and the separated serum was aliquoted, frozen, and stored at −30 °C. The specificity of the method was assessed using serum samples from leftover (surplus) biological material collected for routine laboratory tests which were intended for disposal. The resulting biological material was completely anonymous and contained no information that could be linked to the patient. An example chromatogram of a sample from an anonymous patient treated with AD was shown in Figure 1E.

### 4.6. Sample Preparation

A graphical presentation of the sample preparation procedure is shown in Figure 3.

#### 4.6.1. AD Procedure

Firstly, 200 µL of serum was transferred into a 15 mL Pyrex screw cap glass tube and 25 µL of CAL/QC working solution (or methanol in case of patient sample) was added and vortexed for 10 s; then, 20 µL of IS working solution (BEP, 400 ng) was added and vortexed for 10 s, after which 100 µL of 0.5 M KH_2_PO_4_ was added and vortexed as before. Then, 4 mL of hexane was added to the tube and the sample was extracted automatically using a rotary mixer for 6 min. After extraction, the sample was centrifuged for 10 min at 2000× *g* and then transferred to a −30 °C freezer for 1 h. After freezing the aqueous layer, the organic layer was quantitatively transferred by decantation into a 10 mL Pyrex conical glass tube and evaporated in a water bath at 37 °C under a stream of argon. The dry residue was dissolved in 100 μL of methanol, vortexed, and 50 μL aliquot was applied onto the chromatographic column.

#### 4.6.2. PPF/MEX Procedure

A total of 400 µL of serum was transferred into a 15 mL Pyrex screw cap glass tube, first spiked with 25 µL of CAL/QC working solution (or methanol for patient sample) and vortexed for 10 s, next spiked with 20 µL of IS working solution (GAL, 200 ng) and vortexed for 10 s, then 50 µL of 10% Na_2_CO_3_ solution was added and the sample was vortexed for 10 s. Then, 4 mL of diisopropyl ether was added, and the sample was extracted for 4 min using a rotary mixer. After centrifugation (2000× *g*, 10 min, ambient temperature) and freezing at −30 °C for 45 min, diisopropyl ether was transferred quantitatively into a 10 mL Pyrex conical glass tube. Next, 100 µL of 0.01 M HCl was added to the tube and vortexed for 60 s. The sample was then centrifuged for 5 min at 2000× *g*. The organic phase was removed using a water pump. The aqueous phase was evaporated in a water bath (56 °C) under a stream of argon. The dry residue was dissolved in 100 µL of the mobile phase and finally, 50 μL aliquot was applied onto the HPLC column.

### 4.7. Method Validation

The methodology was subjected to multi-element validation for routine therapeutic drug monitoring within the expected concentration ranges for individual analytes. In the absence of a dedicated TDM guideline, in our studies, in selected areas, we referred to the current (from 21 January 2023) European Medicines Agency (EMA) ICH M10 guideline on bioanalytical method validation [20], and since we started our studies in 2022, also to the previous EMA guideline [19]. This reference particularly applies to the adopted percentage acceptance criteria for accuracy and precision. The following parameters were determined: selectivity and specificity, linearity, calibration, range with LLOQ, carry-over, accuracy and precision.

For testing selectivity and specificity, the study was conducted by analyzing 12 different serum samples (blank matrices not containing the tested substances) including one hemolyzed and one highly lipemic sample, prepared according to both given procedures. For proving the linearity of the detection system, the CAL and QC solutions prepared and described in Section 4.4 were applied onto the column in triplicate, in quantities corresponding to the analyte concentration levels in serum. Calibration curves (triplicates) were obtained by analyzing serum samples spiked with the analyte at each of the six tested concentration levels in duplicate. Calibrations were performed separately for both procedures presented in Section 4.6.1 and Section 4.6.2. The extraction efficiency was calculated by comparing the peak areas of the analytes obtained after extraction (during accuracy and precision testing) with the peak areas recorded after applying appropriate volumes of CAL and QC solutions onto the column. LLOQ was described as the lowest analyte concentration that can be quantitatively determined with acceptable precision (RSD ≤ 20%) and accuracy (inaccuracy ≤ 20%) [20]. In accordance with EMA, which defines accuracy as the degree of closeness of the measured value to the nominal value, we calculated the accuracy as follows: Accuracy (%) = (Measured Value/Nominal Value) × 100 (since we presented the accuracy result (Table 2) as the difference from 100%, we used the term inaccuracy, where inaccuracy = accuracy − 100%). Precision is given as the relative standard deviation (RSD) expressed as a percentage: RSD (%) = (Standard Deviation/Mean) × 100. The acceptance criterion for precision is RSD ≤ 15% and for accuracy, inaccuracy ≤ 15%, except for the LLOQ level given above [20].

## 5. Conclusions

The validation parameters met the requirements, proving that the presented simple HPLC-UV methodology for the determination of AD with DEAD, PPF with its two active metabolites—5OHPPF and NDPPF—as well as MEX in human serum can be successfully used for TDM and even in pharmacokinetic studies during antiarrhythmic therapy. This methodology can be suitable for laboratories with basic HPLC equipment as an economical alternative to the LC-MS/MS technique, which can support the implementation of TDM, especially for LMICs.

## Figures and Tables

**Table 1:** Retention times and extraction efficiency of IS candidates for AD determination.

Analyte	Retention Time [min]	Relative Retention Time	Extraction Yield [%]
TAM (*n* = 5)	4.61	0.65	83.9
BEP (*n* = 5)	4.90	0.69	89.9
DRO (*n* = 4)	4.97	0.70	8.2
DEAD (metabolite, *n* = 16)	5.40	0.77	54.9
AD (parent drug, *n* = 16)	6.06	0.86	90.8
L8040 (*n* = 2)	7.06	1.00	88.7

**Table 2:** Precision and accuracy (*n* = 5).

		INTRA-ASSAY			INTER-ASSAY		
Analyte	Level/ Concentration Added [ng/mL]	Concentration Determined (Mean ± SD) [ng/mL]	Precision (RSD) [%]	Inaccuracy [%]	Concentration Determined (Mean ± SD) [ng/mL]	Precision (RSD) [%]	Inaccuracy [%]
AD	LLOQ-20	19.1 ± 1.1	6.0	−4.7	20.8 ± 1.4	6.7	+3.8
	QC-L-60	59.7 ± 1.2	1.9	−0.5	58.4 ± 1.8	3.0	−2.7
	QC-M-1600	1661.9 ± 47.4	2.9	+3.9	1550.4 ± 62.9	4.1	−3.1
	QC-H-3200	3205.2 ± 112.4	3.5	+0.2	3132.3 ± 56.2	1.8	−2.1
	ULOQ-4000	4033.8 ± 34.9	0.9	+0.8	3977.0 ± 38.9	1.0	−0.6
DEAD	LLOQ-20	20.7 ± 2.3	10.9	+3.4	23.6 ± 2.0	8.7	+18.0
	QC-L-60	54.8 ± 1.2	2.1	−8.6	65.7 ± 8.6	13.1	+9.5
	QC-M-1600	1512.7 ± 47.4	3.1	−5.5	1644.2 ± 113.7	6.9	+2.8
	QC-H-3200	3311.3 ± 112.4	3.4	+3.5	3461.5 ± 217.7	6.3	+8.2
	ULOQ-4000	4219.9 ± 34.9	0.8	+5.5	4409.1 ± 335.5	7.6	+10.2
PPF	LLOQ-10	9.8 ± 0.7	6.9	−2.1	11.8 ± 1.3	10.6	+17.9
	QC-L-30	29.9 ± 3.0	10.0	−0.4	30.8 ± 1.7	5.5	+2.7
	QC-M-1600	1602.0 ± 40.3	2.5	+0.1	1584.8 ± 16.1	1.0	−1.0
	QC-H-3200	3243.6 ± 74.1	2.3	+1.4	3304.4 ± 43.4	1.3	+3.3
	ULOQ-4000	4009.4 ± 39.8	1.0	+0.2	4069.8 ± 50.0	1.2	+1.7
5OHPPF	LLOQ-10	10.5 ± 0.5	8.7	+4.8	10.0 ± 0.5	5.0	−0.4
	QC-L-30	28.5 ± 1.7	5.8	−4.9	27.8 ± 1.9	6.9	−7.2
	QC-M-160	150.0 ± 6.0	4.0	−6.3	153.2 ± 2.0	1.3	−4.3
	QC-H-400	391.5 ± 4.5	1.2	−2.1	410.4 ± 12.1	2.9	+2.6
	ULOQ-500	488.9 ± 9.4	1.9	−2.2	510.3 ± 15.5	3.0	+2.1
NDPPF	LLOQ-10	11.3 ± 1.0	8.7	+12.7	10.8 ± 1.0	9.2	+8.1
	QC-L-30	28.2 ± 1.4	5.0	−6.1	25.6 ± 2.2	8.6	−14.6
	QC-M-160	152.7 ± 8.0	5.2	−4.6	155.9 ± 3.2	2.0	−2.6
	QC-H-400	391.0 ± 8.6	2.2	−2.2	410.5 ± 15.6	3.8	+2.6
	ULOQ-500	488.1 ± 18.6	3.8	−2.4	514.2 ± 19.2	3.7	+2.9
MEX	LLOQ-20	18.0 ± 1.5	8.2	−10.2	19.5 ± 1.4	7.3	−2.3
	QC-L-60	61.7 ± 2.9	4.7	+2.8	57.9 ± 2.5	4.4	−3.5
	QC-M-1600	1627.3 ± 29.7	1.8	+1.7	1610.5 ± 20.4	1.3	+0.7
	QC-H-3200	3188.5 ± 78.2	2.5	−0.4	3260.1 ± 47.1	1.5	+1.9
	ULOQ-4000	3991.7 ± 106.5	2.7	−0.2	4075.7 ± 95.8	2.4	+1.9

Inaccuracy = accuracy − 100%; quality controls (QC): low (QC-L), medium (QC-M) and high (QC-H).

**Table 3:** Extraction efficiency (*n* = 5).

**AD**		**DEAD**		**PPF**	
**Level/** **Concentration Added** **[ng/mL]**	**Yield of Extraction** **(Mean ± SD)** **[%]**	**Level/** **Concentration Added** **[ng/mL]**	**Yield of Extraction** **(Mean ± SD)** **[%]**	**Level/** **Concentration Added** **[ng/mL]**	**Yield of Extraction** **(Mean ± SD)** **[%]**
LLOQ-20	86.0 ± 8.3	LLOQ-20	45.4 ± 6.9	LLOQ-10	86.2 ± 5.2
QC-L-60	86.6 ± 4.7	QC-L-60	47.9 ± 6.9	QC-L-30	81.3 ± 9.1
QC-M-1600	84.3 ± 4.4	QC-M-1600	49.3 ± 4.6	QC-M-1600	77.4 ± 4.5
QC-H-3200	85.2 ± 4.2	QC-H-3200	52.6 ± 4.5	QC-H-3200	80.7 ± 3.0
ULOQ-4000	88.0 ± 1.9	ULOQ-4000	54.6 ± 4.9	ULOQ-4000	78.3 ± 4.5
mean ± SD [%]	86.0 ± 1.4		50.0 ± 3.7		80.8 ± 3.4
**5OHPPF**		**NDPPF**		**MEX**	
**Level/** **Concentration added** **[ng/mL]**	**Yield of extraction** **(mean ± SD)** **[%]**	**Level/** **Concentration added** **[ng/mL]**	**Yield of extraction** **(mean ± SD)** **[%]**	**Level/** **Concentration added** **[ng/mL]**	**Yield of extraction** **(mean ± SD)** **[%]**
LLOQ-10	60.6 ± 3.3	LLOQ-10	71.3 ± 5.1	LLOQ-20	78.9 ± 10.9
QC-L-30	60.1 ± 3.3	QC-L-30	63.0 ± 3.8	QC-L-60	83.7 ± 3.4
QC-M-160	64.0 ± 3.4	QC-M-160	57.9 ± 4.4	QC-M-1600	82.2 ± 3.6
QC-H-400	68.7 ± 3.4	QC-H-400	61.9 ± 3.5	QC-H-3200	84.0 ± 4.1
ULOQ-500	67.6 ± 4.2	ULOQ-500	60.4 ± 2.8	ULOQ-4000	82.8 ± 5.7
mean ± SD [%]	64.2 ± 3.9		62.3 ± 5.1		82.3 ± 2.1

## Data Availability

The original contributions presented in this study are included in the article. Further inquiries can be directed to the corresponding author.

## Abbreviations

The following abbreviations are used in this manuscript:

HPLC	High-performance liquid chromatography
UV	Ultraviolet
CYP	cytochrome P450
LC-MS/MS	Liquid chromatography—tandem mass spectrometry
LC-CN	Cyanopropyl derivatized silica phase
EMA	European Medicines Agency
RSD	Relative standard deviation
